# Potato *Stu-miR398b-3p* Negatively Regulates Cu/Zn-SOD Response to Drought Tolerance

**DOI:** 10.3390/ijms24032525

**Published:** 2023-01-28

**Authors:** Zhiyong Zheng, Jiangwei Yang, Xiaofeng Wang, Ning Zhang, Huaijun Si

**Affiliations:** 1 College of Life Science and Technology, Gansu Agricultural University, Lanzhou 730070, China; 2 State Key Laboratory of Aridland Crop Science, Gansu Agricultural University, Lanzhou 730070, China

**Keywords:** *stu-miR398b-3p*, Cu/Zn SOD, drought resistance, expression analysis

## Abstract

One of the main impacts of drought stress on plants is an excessive buildup of reactive oxygen species (ROS). A large number of ·OH, highly toxic to cells, will be produced if too much ROS is not quickly cleared. At the heart of antioxidant enzymes is superoxide dismutase (SOD), which is the first antioxidant enzyme to function in the active oxygen scavenging system. To shield cells from oxidative injury, SOD dismutation superoxide anion free radicals generate hydrogen peroxide and molecule oxygen. Cu/Zn SOD is a kind of SOD antioxidant enzyme that is mostly found in higher plants’ cytoplasm and chloroplasts. Other studies have demonstrated the significance of the miR398s family of miRNAs in the response of plants to environmental stress. The cleavage location of potato *stu-miR398b-3p* on Cu/Zn SOD mRNA was verified using RLM-5′RACE. Using the potato variety ‘*Desiree*’, the *stu-miR398b-3p* overexpression mutant was created, and transgenic lines were raised. SOD activity in transgenic lines was discovered to be decreased during drought stress, although other antioxidant enzyme activities were mostly unaltered. Transgenic plants will wilt more quickly than wild-type plants without irrigation. Additionally, this demonstrates that the response of Cu/Zn SOD to drought stress is adversely regulated by potato *stu-miR398b-3p*.

## 1. Introduction

MicroRNAs (miRNAs) are a class of 19–24nt non-coding small RNAs with a strong ability to regulate plant stress. An MiRNA cannot directly contribute. It has to be incorporated into the target mRNA by an enzyme-active RNA-induced silencing complex (RISC). By splicing or inhibiting translation of the target gene’s mRNA in response to stress, it negatively affects the expression of the target genes. More and more miRNAs have been found and examined in many plants since Llave initially found and studied the mechanism of action of *Arabidopsis thaliana* L. miRNAs [1]. Crop plants include *Oryza sativa* L., *Zea mays* L., *Titicum aestivum* L. and *Solanum tuberosum* L. and others have obtained fruitful results in their respective fields [2,3,4,5]. Kirch and colleagues discovered that dryness, excessive salt, and exogenous ABA could also activate the cold-induced transcription miRNA CI7 in potato tubers [6]. Deep sequencing was employed by Zhang et al. to find the miRNAs connected to the potato drought [7]. MiR398 can control how plants react to oxidative stress, water shortage, salt stress, abscisic acid stress, UV stress, copper and phosphate deprivation, among other stresses, according to research by Zhu et al. [8]. Numerous studies have demonstrated that miRNAs are essential regulators of plant physiological and biochemical processes, including appropriate growth and development. Therefore, using miRNAs to genetically increase stress tolerance in crop cultivars is particularly promising.

Since potatoes are a shallow root crop that prefer the cold, their yield will decrease significantly if the ground temperature rises above 25 °C. Potato yield was severely impacted by drought stress, which also resulted in significant losses. Recently, numerous advances have been made in the understanding of potato drought tolerance [9]. Plant cells’ chloroplasts, mitochondria, and plasma membranes all create reactive oxygen species (ROS) during the transport of electrons. These ROS are often produced by plants as part of their physiological metabolism. When ROS are abnormally generated under drought stress and are not broken down, a significant amount of ·OH is created by the Fenton or Haber–Weiss reaction. These ·OH are the free radicals that harm cells the greatest. At the heart of antioxidant enzymes, superoxide dismutase (SOD) is the first enzyme to take part in the scavenging response of ROS. Peroxidase (POD) and catalase (CAT) are further crucial antioxidant enzymes. They are crucial in preserving the equilibrium of intracellular ROS and avoiding membrane system deterioration. According to the study of Xu et al., the transgenic cassava plants with enhanced expressions of cytoplasmic MeCu/Zn SOD and peroxisome MeCAT1 had significantly better water retention capacity than non-transgenic plants at 30 days after water deprivation [10]. Tian et al. have shown that SOD1 in *Verticillium dahlia* is not essential for development or survival, but it is involved in the detoxification of intracellular ROS and extracellular ROS produced by hosts. [11]. The result from analysis of the expression patterns of the Cu/Zn SOD genes from *Solanum lycopersicum* and *Arabidopsis thaliana* under various conditions, the Cu/Zn SOD gene family has the potential for functional divergence and may be essential for ROS scavenging in plants as a result of a variety of stresses [12]. Studies have demonstrated that Cu/Zn SOD is crucial for preserving the balance of intracellular ROS.

In addition to participating in related regulation in saline-alkaline, drought, heavy metals, and other conditions, miR398s can play a variety of roles in the stress resistance process of many plants. According to Guan et al., in order to withstand heat stress, target genes CSD1, CSD2, and CCS (copper chaperone) downregulate the expression of mature miR398 in *Arabidopsis thaliana* [13]. According to Chen et al., miR398 was down-regulated in transgenic *Arabidopsis thaliana*, while its two target genes, CSD1 and CSD2, were up-regulated, increasing the plant’s ability to withstand freezing temperatures [14]. *miR398a/b* expression was down-regulated in the roots and buds of pea (*Pisum sativum* L.) under water shortage circumstances, while CSD1 expression was up-regulated, which contributed to pea resilience to water deficit stress [15]. Additionally, miR398 can control the *PetE2* gene, which links copper levels in *Arabidopsis* to the production of three important chloroplast copper proteins, plastid protein, CCS1 and CSD2, and other proteins [16]. To withstand the stress of nutrient imbalance, copper deficiency in *Arabidopsis* stimulates the production of miR397, miR398, miR408, and miR857 and controls the down-regulation of copper protein [17]. The studies mentioned above have demonstrated that miR398 primarily affects plant stress resistance by controlling the expression of its target genes CSD1, CSD, CCS1, etc. It was also predicted by software that the target genes of *miR398-3p* have functions related to plant stress resistance, especially Cu/Zn SOD.

## 2. Results

### 2.1. Secondary Structure Analysis of Stu-miR398b-3p Precursor Sequence

The precursor and mature sequences of miRNAs were obtained from miRbase (http://www.mirbase.org/). The secondary structure of the *stu-miR398b-3p* precursor sequence (MI0025983) was analyzed by online software RNAfold Web-Sever. It showed that *stu-miR398b-3p* could form a stable stem-loop structure. The free energy of its thermodynamic ensemble was −114.51 kcal/mol, the frequency of MFE structure in the ensemble was 0.00%, and the overall diversity was 70.61, which was consistent with the basic characteristics of miRNA characteristic structure.

### 2.2. Prediction and Validation of Stu-miR398b-3p Target Genes

**Figure 1:**
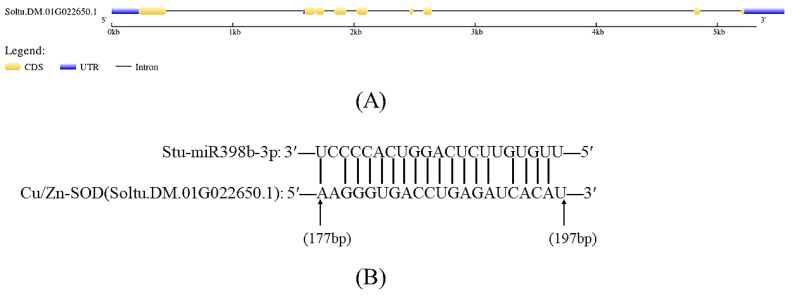
Structure and binding site of *stu-miR398b-3p* target gene. (**A**) Structure of *stu-miR398b-3p* target gene. (**B**) *Stu-miR398b-3p* target gene binding site. Soltu.DM.01G022650.1 is the sequence ID of the *stu-miR398b-3p* target gene in PGSC. The bases at position 178 are not complementary and the bases at positions 192 and 197 are identical.

The mature sequence of *stu-miR398b-3p* was input into the plant miRNA target gene online prediction software psRNATarget, and 116 potato gene sequence numbers containing target gene binding sites were obtained. The potato database PGSC was searched one by one to obtain the sequence structure of the target gene Cu/Zn SOD gene (Soltu.DM.01G022650.1, PGSC0003DMT400027651) (Figure 1A). The binding site of *stu-miR398b-3p* to Cu/Zn SOD gene was determined by RLM-5′RACE amplification of the nucleic acid sequence near the Cu/Zn SOD mRNA fracture (Figure 1B).

### 2.3. Bioinformatics Analysis of Stu-miR398b-3p Target Genes

The predicted secondary structure of the *stu-miR398b-3p* target gene protein shows that the protein has a total of 152 amino acid sequences, of which 8 are α-helix structures, 48 belong to extended chains, and 14 are β-turn structure; the remaining 82 are random coil sequences. The tertiary structure was predicted, and it was found that the protein structure of *stu-miR398b-3p* target gene was highly similar to that of Cu/Zn-SOD in various organisms. By analyzing the physicochemical properties, the molecular formula of the protein is C_653_H_1042_N_194_O_221_S_4_, which is composed of 2114 atoms and has a molecular weight of 15274.86. It consists of 152 amino acids, including 16 negatively charged residues and 8 positively charged residues. The isoelectric point was 5.28, and the total average hydrophilicity was −0.191. Analysis of the transmembrane structure shows that there is no fluctuation or junction between the inner and outer membranes, and the protein does not have a transmembrane structure. It has a total of 9 phosphorylation sites, including 6 serine phosphorylation sites, 3 threonine phosphorylation sites, and no tyrosine phosphorylation sites. Its interaction network has a total of 11 nodes, a total of 43 interaction lines, an average node degree of 7.82, and an average local clustering coefficient of 0.921. According to the *stu-miR398b-3p* target gene protein interaction map, there are 7 interacting proteins with SOD functions, PGSC0003DMT400027651, PGSC0003DMT400009994, PGSC0003DMT400001102, PGSC0003DMT400070920, PGSC0003DMT400046236, PGSC0003DMT400042937, PGSC0003DMT400013447. One of them, PGSC0003DMT400001102, and *stu-miR398b-3p* target gene protein belonged to the Cu/Zn SOD family. Another three, PGSC0003DMT400075611, PGSC0003DMT40000398, PGSC0003DMT400025653, proteins are hydrogen peroxide isoenzymes. There is one protein, PGSC0003DMT400085026, as the A1 member, which exists in the aldehyde dehydrogenase family 7.

### 2.4. Stu-miR398b-3p Construction of a miRNA Expression Vector

Primers I, II, III and IV (Table 1) of *stu-miR398b-3p* were designed with WMD3 using the universal vector pRS300 as a template. The first round of amplification was performed by nested PCR reactions, resulting in a 272bp 5′-arm fragment, a 17l bp central loop, and a 298 bp 3′-arm fragment. The a, b and c fragments recovered from glue were used as templates for the second round of PCR amplification. Using universal primers, A and B, as upstream and downstream primers, the d fragment with a length of 701 bp was amplified. [18]

**Figure 2:**
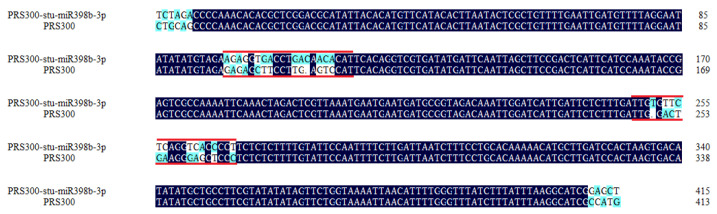
Sequencing results of positive bacteria were compared with PRS300 vector sequence. Navy blue represents the same bases of the two sequences. The sky blue represents the different bases of the two sequences. The red lines represent the insertion position of *stu-miR398b-3p*.

The d fragment was linked to the PMD18-T cloning vector, and then transferred into *E. coli* DH5α competent cells after sequencing verification. A single positive clonal colony was selected for PCR detection and sent to the company for sequencing. The d fragment was successfully imported by comparing the sequence obtained by sequencing with the PRS300 vector sequence (Figure 2). The plasmid was extracted from the correct bacterial fluid and ligated into pBI121 vector by double restriction endonuclease *Xba* I (5815 bp) and *Sac* I (5179 bp). The vector was named pBI121-*stu-miR398b-3p*.

### 2.5. Potato Genetic Transformation and Identification of Transgenic Plants

**Figure 3:**
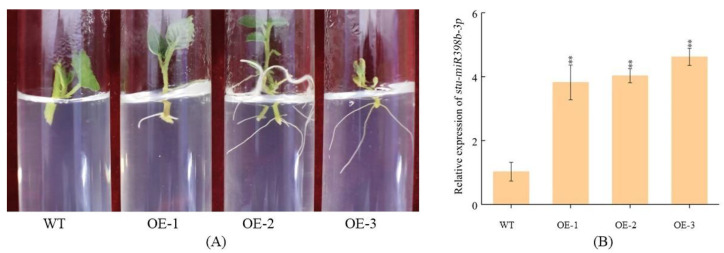
(**A**) Rooting selection of *stu-miR398b-3p* overexpression plants. After 7 days in the selective medium, OE-1, OE-2, and OE-3 rooted while WT did not. The test tube plantlets of 9 WT lines with biological and technical duplication did not root in the rooting selection medium. The test tube plantlets of 9 WT lines with biological replicates and technical replicates did not root in selection medium. The 9 test-tube plantlets of the 3 OE lines all gradually rooted within 7 days, and there were no plants that did not root. (**B**) Identification of *stu-miR398b-3p* overexpression lines by qRT-PCR. Three independent biological samples of three technical replicates for qRT-PCR analysis. Each column represents the mean values ± SE (*n* = 3; ** *p* < 0.01). WT: wild type; OE-1, OE-2 and OE-3: *stu-miR398b-3p* overexpression lines.

Using potato as the material, the pBI121-stu-miR398b-3p overexpression vector was introduced into potato plants by competent *Agrobacterium* infection for tissue culture. After 15 days of culture in differentiation medium, the potato slices turned green, formed callus, and started shooting. When the buds were 1 to 2 cm long, they were cut and transplanted into 3% MS medium supplemented with kanamycin (Kan) for culture. The pBI121-stu-miR398b-3p overexpression transgenic lines were obtained by selecting the rooting lines for propagation and culture (Figure 3A).

DNA was extracted from the rooting lines, and the target gene was amplified by PCR. The results showed that the bands were bright and clear, which proved that the transgenic lines contained the target fragment. The leaves of non-transgenic and screened overexpressed lines were used as samples to extract RNA, reverse transcribed to obtain cDNA, and the transgenic plants were identified by quantitative real-time PCR (qRT-PCR). The results showed that the expression levels of *stu-miR398b-3p* in the transgenic lines OE-1, OE-2 and OE-3 were 3.8, 4.0 and 4.6 times higher than those in the WT lines, respectively (Figure 3B).

### 2.6. Overexpression of Stu-miR398b-3p Inhibits the Expression of Its Target Genes

**Figure 4:**
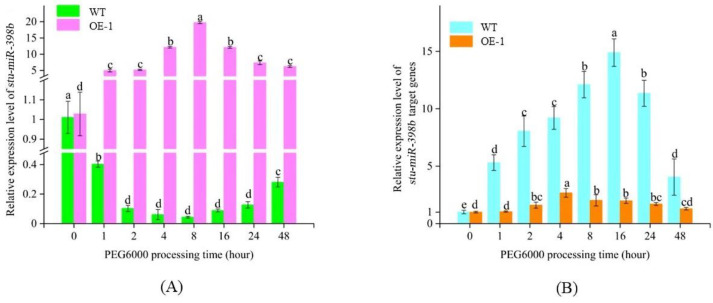
Expression analysis of *stu-miR398b-3p* and its target genes by qRT-PCR. (**A**) *Stu-miR398b-3p* expression in OE-1 and WT lines. (**B**) expression of *stu-miR398b-3p* target genes in OE-1 and WT lines. Three independent biological samples of three technical replicates for qRT-PCR analysis. Each column represents the mean values ± SE (*n* = 3). The letters indicate significant difference according to one-way ANOVA corrected by Dunnett (*p* < 0.05).

The expression levels of *stu-miR398b-3p* and its target genes in the samples stored at −80 °C were determined by qRT-PCR (Figure 4). It showed that the expression of *stu-miR398b-3p* in wild-type plants decreased significantly with the change in treatment time under drought stress, while the expression of *stu-miR398b-3p* overexpression transgenic plants increased significantly. The expression of the *stu-miR398b-3p* target gene in wild-type plants increased significantly with time in response to drought stress. However, in the *stu-miR398b-3p* overexpressing transgenic plants, the expression of target genes of *stu-miR398b-3p* was much lower than that of wild-type plants, although it still increased. In conclusion, the overexpression of *stu-miR398b-3p* can inhibit the expression of target genes and reduce the drought resistance of plants.

### 2.7. Stu-miR398b-3p Negatively Regulates Drought Tolerance of Potato

#### 2.7.1. Natural Drought Treatment

**Figure 5:**
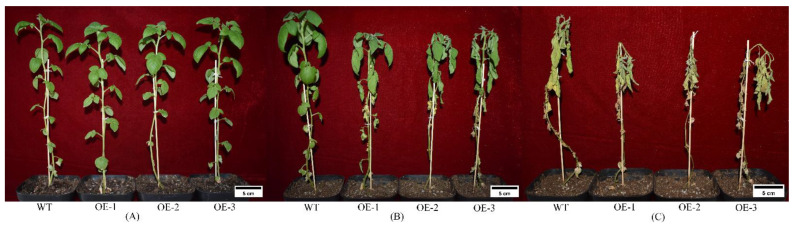
Natural drought treatment of *stu-miR398b-3p* overexpression lines. (**A**) On day 0, there was no significant difference in the normal growth status of potato WT lines and OE lines. (**B**) On the 12th day, potato WT and OE plants showed phenotypic differences. The color of the leaves of the WT plants was bright, and the color of the leaves of the OE plants was dim. The leaves of WT plants were firm, and the leaves of OE plants were wilting. (**C**) On the 21th day, the hue is gloomy, the leaves are fragile and drooping, and WT plants are dying. Plants of OE were died, and their leaves turned yellow and dried out. Three independent biological samples with three technical replicates were used for drought resistance analysis. A total of 36 pots of potato plants were planted, including 9 pots of WT lines and 9 pots of each OE line. And all plant phenotypic changes are consistent with the above.

**Figure 6:**
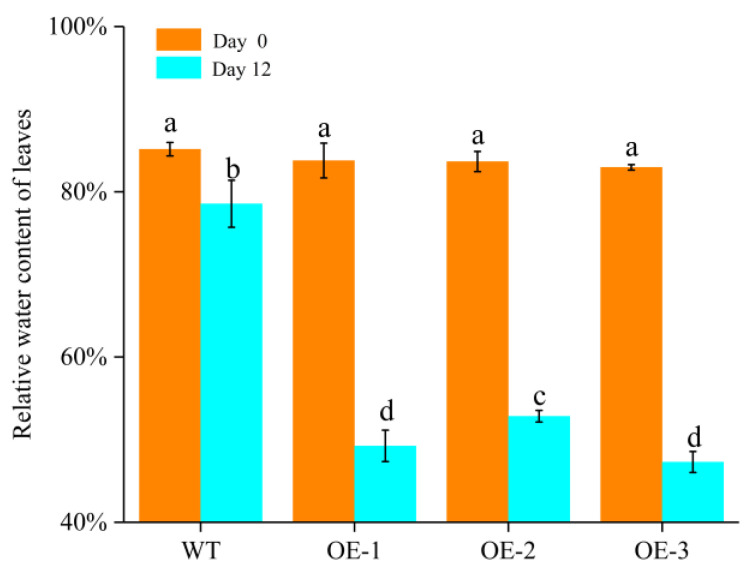
*LRWC* changes under natural drought. Three potato plants were selected from each strain, and three groups of repeats were taken from each plant, and the 3–5 leaves were selected from the top to the bottom. Each column represents the mean values ± SE (*n* = 3). The letters indicate significant difference according to one-way ANOVA corrected by Dunnett (*p* < 0.05).

The drought tolerance of potato *stu-miR398b-3p* overexpression lines and wild-type lines was significantly different (Figure 5). The potato *stu-miR398b-3p* overexpression lines grew normally for the first 6 days, but after that point the leaves started to gradually dry up until they eventually withered and died on the 18th day. The wild-type strain grew normally for the first 12 days. At 15 days, the leaves started to indicate a modest degree of dehydration, and after 21 days, the strain died. In comparison to wild-type lines, the drought tolerance of potato *stu-miR398b-3p* overexpression lines was significantly reduced. The leaf relative water content (*LRWC*) of *stu-miR398b-3p* overexpression lines decreased much more than that of wild-type lines in the first 12 days (Figure 6). Under drought stress, the decreasing rate of *LRWC* in wild-type leaves was slow, the decreasing amplitude was relatively small, and the water retention capacity was strong. The *LRWC* of *stu-miR398b-3p* overexpression lines decreased rapidly, and the water retention ability was poor. It indicated that under natural drought conditions, the drought tolerance of wild-type lines was stronger than that of *stu-miR398b-3p* overexpression lines.

#### 2.7.2. PEG6000 Simulated Drought Treatment

**Figure 7:**
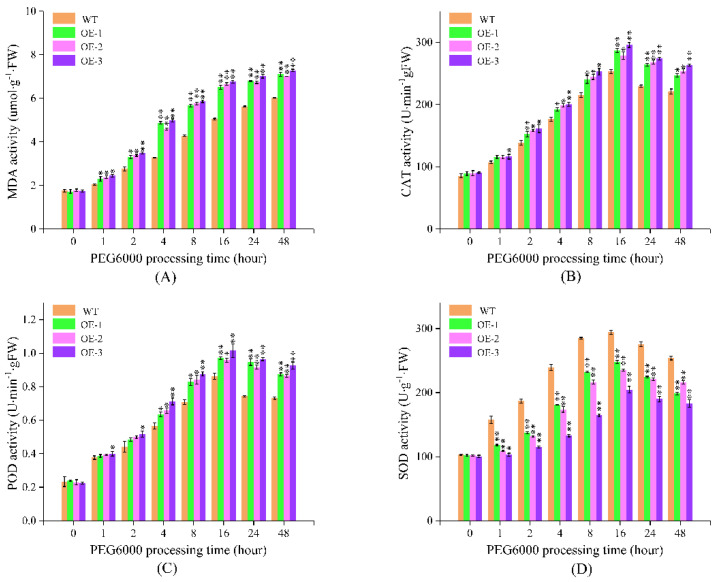
Determination of activity of MDA, CAT, POD and SOD after irrigation with 20% PEG6000. (**A**) The content of MDA, (**B**) the enzyme activity of CAT, (**C**) the enzyme activity of POD, (**D**) the enzyme activity of SOD. Three independent biological samples of three technical replicates for RT-PCR analysis. Each column represents the mean values ± SE (*n* = 3; * *p* < 0.05; ** *p* < 0.01).

Under simulated drought conditions, the activities of malondialdehyde (MDA), CAT, POD and SOD in potato plants showed an upward trend, reached the peak at 16 h, and then fluctuated slightly (Figure 7). The *stu-miR398b-3p* overexpression lines and wild-type plants showed considerable or extremely significant differences in the same period after the simulated drought treatment. At the same time after treatment, wild-type plants’ MDA, CAT, and POD activities were lower than those of *stu-miR398b-3p* overexpression lines, although SOD activity was the opposite.

## 3. Discussion

### 3.1. In Vivo Cleavage of Cu/Zn SOD mRNA Mediated by Stu-miR398b-3p

Cu/Zn SOD catalyzes the disproportionation of superoxide anion radicals to produce oxygen and hydrogen peroxide, which plays a vital role in the balance of oxidation and antioxidation. Plant SOD antioxidant response to drought stress has also been widely studied. Here, we predicted the target gene of potato *stu-miR398b-3p* in chloroplast (Cu/Zn SOD gene, Soltu.DM.01G022650.1). The RLM-5′RACE experiment also effectively verified the binding site of *stu-miR398b-3p* to Cu/Zn SOD. The qRT-PCR also showed that the expression levels of *stu-miR398b-3p* and Cu/Zn SOD showed an opposite trend under drought stress, which was similar to the expression patterns between known miRNAs and target genes. In addition, overexpression of *stu-miR398b-3p* inhibited the expression of Cu/Zn SOD in transgenic lines overexpressing *stu-miR398b-3p*. In summary, this proves that Cu/Zn SOD is the target gene of *stu-miR398b-3p* and is cleaved by *stu-miR398b-3p* in vivo.

### 3.2. Stu-miR398b-3p Negatively Regulates Drought Tolerance of Potato

Drought stress has been seriously affecting the yield and quality of crops. MiRNAs have always been closely related to plant drought stress. In general, miRNAs negatively regulate target genes; that is, when miRNAs are up-regulated, the expression of target genes will be inhibited or the target gene products will be decomposed, leading to the down-regulation of target gene expression, and vice versa [19]. MiRNAs can regulate the expression of target genes to respond to the adverse response of drought stress on plants and improve the survival ability of plants under drought environment. Currently, known miRNAs related to drought stress include miR156, miR160, miR167, miR169, miR171, miR172, miR319, miR394, miR396, miR397 and miR398 [20,21,22,23,24,25,26,27,28]. Zhu et al. introduced the important role of miR398 in plant stress response and discussed the miR398 gene regulatory network [9]. MiR398 affects the activity of CSD and cytochrome C oxidase subunit V to regulate oxidative stress and enzymes involved in respiration. Dugas and Bartel found that CSD mutants with miR398 resistance expressed CSD1 and CSD2 mRNA up-regulated, but CSD1 and CSD2 protein accumulation decreased [29]. This suggests that miR398 may inhibit the translation of CSD1 and CSD2 mRNA, thereby negatively regulating the target. In the study of the model plant *Arabidopsis thaliana*, miRNA398 was found to regulate the CSD gene in response to oxidative stress [30]. MiR398 was also found to be involved in the regulation of CSD decomposition of superoxide root ions in rice, tobacco, maize and other plants. Therefore, miR398 in potato should have the same or similar function [30,31,32,33].

MDA is a measure of the damage caused by membrane lipid peroxidation, which can inadvertently reflect the severity of the damage to potatoes under drought stress. SOD, POD, and CAT have a role in controlling potato ROS and are crucial markers of potato drought resistance. Potato plants under drought stress accumulated excessive ROS and developed toxicity. The overexpression of *stu-miR398b-3p* in *stu-miR398b-3p* overexpression lines decreased SOD activity. SOD activity was much reduced in *stu-miR398b-3p* overexpression lines than in wild type. The capacity of SOD’s ROS degradation pathway diminished, which led to a reduction in the ability of potato plants to withstand drought. Therefore, compared to potato wild-type lines, the MDA concentration of *stu-miR398b-3p* overexpression lines was greater. Along with SOD, POD and CAT can also breakdown ROS. The rise in POD and CAT activity will result from the decrease in SOD activity. It explained why, during a drought treatment, the activity of POD and CAT in *stu-miR398b-3p* overexpression lines was always marginally higher than that in wild type. *Stu-miR398b-3p* adversely regulated the expression of Cu/Zn SOD in response to drought stress, as demonstrated by the results of MDA, CAT, POD, and SOD activities in wild-type and overexpressed *stu-miR398b-3p* lines.

## 4. Materials and Methods

### 4.1. Vector and Reagents

The pRS300 vector, expression vector pBI121 (CaMV 35S promoter, resistance marker Kanamycin) and *Agrobacterium tumefaciens* GV3101 are preserved by Genetic Laboratory, College of Life Science and Technology, Gansu Agricultural University (Lanzhou, China). Rapid Plant Genomic DNA Isolation Kit, SanPrep Column Plasmid Mini-Preps Kit, DiaSpin DNA Gel Extraction Kit, One Step qRT-PCR Probe Kit and SuperReal PreMix Plus (SYBR Green) were purchased from Sangon Biotech (Shanghai, China) Co., Ltd. TRNzol Universal Reagent and MiRNA cDNA First Strand Synthesis Kit were purchased from Accurate Biology (Hunan, China) Co., Ltd. pMD18-T vector, restriction endonuclease, T4 DNA ligase, 2 × EasyTaq^®^ PCR SuperMix and *Escherichia coli* strain DH5α were purchased from TransGen Biotech (Beijing, China) Co., Ltd. Takara EX Taq(#RR902A), SMART ™ RACE cDNA Amplification Kit, Thiobarbituric acid (TBA), Nitro-Blue Tetrazolium (NBT), 2-methoxyphenol and other reagents were purchased from Takara Bio (Dalian, China) Co., Ltd. Primer synthesis and sequencing was completed by Lanzhou Tqgene Gene Biotechnology Co., Ltd.

### 4.2. Potato Materials and Drought Treatments

Plant material for this experiment came from the Genetic Laboratory of the College of Life Science and Technology, Gansu Agricultural University, and was a drought-sensitive potato variety “Desiree”. After 30 days of incubation in 3% MS solid medium, “Desiree” wild-type and *stu-miR398b-3p* overexpression transgenic plants were transplanted into POTS (vegetative soil:vermiculite = 1:1). The plants were given a drought treatment after 30 days of incubation in POTS.

Treatment for natural drought: The experimental group was chosen from three transgenic lines and three wild-type lines with the same growth pattern. The watering was halted, the natural drought experiment was initiated, and the experiment was recorded every three days when the relative water content of the soil, as determined by the soil moisture tachometer, was between 60% and 80% (the soil was in moderate drought).

Three transgenic lines and three wild-type lines with the identical growth trend were chosen to make up the experimental group for the PEG6000 drought treatment. The plants were watered thoroughly with 20%PEG6000 when the relative water content of the soil was between 60% and 80% (the soil was experiencing a mild drought), and the third to fourth leaves under the leaf crown were taken as samples in liquid nitrogen after 0 h, 1 h, 2 h, 4 h, 8 h, 16 h, 24 h, and 48 h watering, and then stored at −80 °C.

### 4.3. Prediction of Stu-miR398b-3p Target Gene

The precursor and mature sequences of miRNAs were obtained and analyzed according to miRbase (http://www.mirbase.org/). The online software RNAfold Web-Sever was used to analyze the precursor sequence of *stu-miR398b-3p*. Plant miRNA target genes online prediction software psRNATarget “http://plantgrn.noble.org/v1_psRNATarget/ (accessed on 8 May 2022)” was used to predict *stu-miR398b-3p* target genes. The target gene and protein sequences of potato *stu-miR398b-3p* were downloaded from the database PGSC (PGSC *S. tuberosum* group *phureja* DM1-3).

### 4.4. RLM-5′RACE Method to Verify the Target Gene of Stu-miR398b-3p

The RLM-5′RACE method is the most commonly used method to verify miRNA target genes in plants. The cleavage effect of *stu-miR398b-3p* on target genes was verified by cutting the end of Cu/Zn SOD mRNA product and amplification sequencing. Two gene-specific nested primers (outer primer: 5′-GCTGCATGTCAACAGGACCACGTGGTCCTGTTGACATGCAGC-3′; inner primers: 5′-GGTATCACCAAGGGCATGGACATGGAAG-3′) were designed downstream of the possible splicing site of potato Cu/Zn SOD gene by *stu-miR398b-3p*. Then, separately with upstream SMART II A usual joint outer primers (5′-ctaatacgactcactatagggcAAGCAGTGGTATCAACGCAGAGT-3′) and inner primers (5′-ctaatacgactcactatagggc-3′) are two rounds of nested PCR amplification. Total RNA was extracted from the whole “*Desiree*” tissue culture seedling and the first-strand cDNA with SMART II A linker was reverse transcribed using SMART™ RACE cDNA amplification Kit. After 10-fold dilution of cDNA, the first round of nested PCR was performed using Takara EX Taq, and 1μL of the first-round product was used as a template for the second round of nested PCR. After the second round of PCR products were electrophoresed on agarose gels, the target fragments were cut, recovered and purified, and ligated into the pMD18-T cloning vector. Then, DH5α competent *Escherichia coli* was transformed, coated with medium plates, and cultured at 37 °C overnight. Ten monoclonal clones were selected from each plate for sequencing [34].

### 4.5. Bioinformatics Analysis of Stu-miR398b-3p Target Genes

SOPMA “http://npsa-pbil.ibcp.fr/cgi-bin/npsa_automat.pl?page=npsa_sopma.html (accessed on 10 May 2022)” software was used to predict the secondary structure of the *stu-miR398b-3p* target gene protein, using the SWISS-MODEL “https://swissmodel.expasy.org/ (accessed on 10 May 2022)” predict *stu-miR398b-3p* target genes protein tertiary structure. The online tool ProtParam “https://web.expasy.org/protparam/ (accessed on 10 May 2022)” can analyze the physicochemical properties of the target gene protein of *stu-miR398b-3p*. The online software TMHMM “http://www.cbs.dtu.dk/services/TMHMM/ (accessed on 11 May 2022)” was used to predict the transmembrane structure of the *stu-miR398b-3p* target gene protein. NetPhos 3.1 Server “http://www.cbs.dtu.dk/services/NetPhos/ (accessed on 11 May 2022)” was used to predict the protein phosphorylation sites of *stu-miR398b-3p* target genes. The online analysis software STRING “https://string-db.org/cgi/input.pl (accessed on 11 May 2022)” was used to predict the protein interaction network of the *stu-miR398b-3p* target gene proteins.

### 4.6. Construction of Stu-miR398b-3p Artificial miRNA Expression Vector

The correct plasmid pT-amiR398b-3p was selected and digested with *Xba* I and *Sac* I, and about a small 512 bp fragment. At the same time, pBI121 plasmid was digested with *Xba* I and *Sac* I to recover the large fragment. The pT-amiR398b-3p recovery fragment and the large fragment recovered from the pBI121 plasmid were mixed at a DNA molar ratio of 3:1, and 1 μL of T4 DNA ligase and 1 μL of 2 × T4 DNA ligase Buffer was added. The volume of sterile double distilled water was added to 10 μL. The sample was mixed and centrifuged instantaneously for 10 s, and then the reaction system was placed in a PCR instrument at 25 °C for 30 min. Then 10 μL ligation product was transferred into competent *E. coli* DH 5α by the heat shock method and coated on a plate containing Kan-resistant LB medium for positive screening. It was named pBI121-*stu-miR398b-3p* after a series of verifications such as bacterial PCR, enzyme digestion and sequencing.

### 4.7. Potato Genetic Transformation and Identification of Transgenic Plants

Induction of microtuber: 6% MS solid medium was used to inoculate potato variety “*Desiree*” in batches. After 30 days of growth at 20,000 lx, light 16 h/dark 8 h, and 24 °C, all plants were cultured in the dark for 60 days to obtain induced potatoes about 1cm in diameter.

The pBI121-*stu-miR398b-3p* plasmid was transformed into *Agrobacterium* competent cells by the freeze-thaw method. The pBI121-*stu-miR398b-3p* overexpression vector was introduced into potato tubers for tissue culture. When the callus germinated, 1–2 cm buds were cut and transferred into the sieve root medium with kanamycin. Rooting lines were selected for transgenic identification. The DNA of the rooting strain was extracted and the target gene was amplified by PCR. The electrophoretogram was used to identify whether the rooting strain contained the target fragment. RNA was extracted from the leaves of wild type and root strain, and cDNA was obtained by reverse transcription. The transgenic plants were identified by qRT-PCR.

### 4.8. Drought Resistance Analysis of Transgenic Plants

In order to study the impact of *stu-miR398b-3p* overexpression on potato drought tolerance, it was necessary to compare the drought tolerance of wild-type plants with potato *stu-miR398b-3p* overexpression lines. In transgenic and wild-type potato lines subjected to PEG6000 simulations of drought, the expression levels of *stu-miR398b-3p* and target genes were evaluated, and the drought resistance of overexpressed plants was compared with the effects of natural drought.

The concentration of MDA was assessed using the TBA technique. The NBT photochemical reduction technique was used to measure SOD activity. The guaiacol method was used to assess the activity of POD. The UV absorption method was used to assess the CAT activity. Potato *LRWC* was determined by the saturation weighing method. Three duplicates of each experiment were carried out. Data were entered and simple calculations were carried out in Microsoft Excel (Microsoft PLC., Redmond, WA, USA). For the significance analysis, SPSS atistics version 17.0 (SPSS Inc., Chicago, IL, USA) was employed. By using Origin 2017 (QriginLab Inc., Northampton, MA, USA), the drought resistance of potato wild-type and *stu-miR398b-3p* overexpressed plants was compared and examined.

## 5. Conclusions

In conclusion, the *stu-miR398b-3p* target gene and cleavage site in potato was identified and confirmed using RLM-5′RACE. Using amiRNA technology, the *stu-miR398b-3p* overexpression plants were created. The qRT-PCR was used to compare and examine the expression levels of *stu-miR398b-3p* and its target genes in the leaves of wild-type and transgenic plants under drought stress. The expression of target genes was shown to be strongly reduced by *stu-miR398b-3p* overexpression. Additionally, during drought stress, SOD activity in wild-type potato lines was much lower than that in transgenic lines, and MDA activity was the opposite, showing that the lines that overexpressed the *stu-miR398b-3p* were less tolerant to drought. These results indicated that overexpression of *stu-miR398b-3p* could inhibit the expression of the Cu/Zn SOD dismutase gene and reduce the drought tolerance of potato seedlings under drought stress.

## Figures and Tables

**Table 1:** Primers for *stu-miR398b-3p*.

Primers	Primer Sequences (5′–3′)
I miR-s	gaTTGTGTTCTCAGGTCACCCCTtctctcttttgtattcc
II miR-a	gaAGGGGTGACCTGAGAACACAAtcaaagagaatcaatga
III miR*s	gaAGAGGTGACCTGACAACACATtcacaggtcgtgatatg
IV miR*a	gaATGTGTTGTCAGGTCACCTCTtctacatatatattcct

## Data Availability

Not applicable.
